# Plant Responses to Abiotic Stress Regulated by Histone Deacetylases

**DOI:** 10.3389/fpls.2017.02147

**Published:** 2017-12-15

**Authors:** Ming Luo, Kai Cheng, Yingchao Xu, Songguang Yang, Keqiang Wu

**Affiliations:** ^1^Guangdong Provincial Key Laboratory of Applied Botany, Key Laboratory of South China Agricultural Plant Molecular Analysis and Genetic Improvement, South China Botanical Garden, Chinese Academy of Sciences, Guangzhou, China; ^2^College of Life Science, Institute of Plant Biology, National Taiwan University, Taipei, Taiwan

**Keywords:** histone deacetylation, HDACs, abiotic stress, autophagy, protein complexes

## Abstract

In eukaryotic cells, histone acetylation and deacetylation play an important role in the regulation of gene expression. Histone acetylation levels are modulated by histone acetyltransferases and histone deacetylases (HDACs). Recent studies indicate that HDACs play essential roles in the regulation of gene expression in plant response to environmental stress. In this review, we discussed the recent advance regarding the plant HDACs and their functions in the regulation of abiotic stress responses. The role of HDACs in autophagy was also discussed.

## Introduction

In eukaryotes, DNA is packaged into chromatin with the core histone proteins including histone H2A, H2B, H3, and H4. Histone proteins are subjected to various post-translational modifications including acetylation, methylation, phosphorylation, ubiquitination, sumoylation, and ADP ribosylation (Berger, 2007). Histone acetylation plays fundamental roles in the regulation of gene expression in many aspects of biological processes. Histone acetylation levels are reversibly regulated by histone acetyltransferases (HATs) and histone deacetylases (HDACs). HATs can acetylate the specific lysine residues on N-termini of histone H3 and H4, resulting in activation of gene transcription. On the contrary, HDACs link to transcriptional repression and gene silencing through deacetylation of lysine residues. Chromatin structure can be affected by altering the acetylation of the histone tails by HATs and HDACs through mechanism of the effect of so-called “open/closed DNA” for transcription. HDACs lack intrinsic DNA-binding activity and are recruited to target genes via their association with transcriptional factors as well as their incorporation into large multiprotein transcriptional complexes. Furthermore, removing acetyl groups from histone by HDACs causes the chromatin more tightly packed and leads to a reduced accessibility for transcription factors to bind to the DNA, resulting in transcriptional repression. Additionally, the removal of acetyl group from N- terminal tails of histone may also change the structure of chromatin and modulate the interaction of histone tails with its interacting partners. Multiple specific lysine sites of histone acetylation have been identified, including histone H2AK5, H2B (K5, K12, K15, K20), H3 (K4, K9, K14, K18, K23, K27), and H4 (K5, K8, K16, K12, K16) (Peterson and Laniel, 2004). In general, H2A and H2B acetylation is associated with gene activation. Similarly, the combination of H3K9 and H3K14 acetylation is also linked to transcriptional activation. In addition, H4K5 acetylation is connected with transcriptional activation, histone deposition and DNA repair (Peterson and Laniel, 2004).

## HDACs in Plants

Histone deacetylases are highly conserved in many organisms, including fungi, animals, and plants (Ekwall, 2005). Multiple HDACs have been identified and characterized in plants (Hollender and Liu, 2008; Hu et al., 2009; Aquea et al., 2010; Liew et al., 2013; Zhao L. M. et al., 2015; Peng et al., 2017). Eighteen HDACs identified in *Arabidopsis* can be grouped into three main families including the RPD3/HDA1 family (homologous to yeast RPD3), Sir2 family (homologous to yeast Sir2), HD family (plant specific HDACs). The RPD3 family HDACs can be further divided into three classes by sequence similarity: class I (HDA6, HDA7, HDA9, and HDA19), class II (HDA5, HDA15, and HDA18), and class III (HDA8, HDA10, HDA14, and HDA17). HD2 proteins are plant specific HDACs, which was firstly identified in maize by Lusser et al. (1997). There are four HD2 type HDACs including HD2A, HD2B, HD2C, and HD2D in *Arabidopsis*. Sir2 family HDACs are nicotinamide adenine dinucleotide (NAD) dependent HDACs and there are two members of Sir2-like HDACs, SIR1 and SIR2. In *Arabidopsis*, HDACs play vital roles in plant development and in responses to various stresses (Hollender and Liu, 2008; Luo et al., 2012a,d, 2015; Han et al., 2016; Yu et al., 2017).

The rice genome also contains 18 HDACs, including 14 members of the RPD3/HDA1 family, two members of the Sir2 family and two members of the HD2 family (Hu et al., 2009). Several rice HDACs were reported to function in responses to various abiotic stress (Hu et al., 2009; Zhao J. H. et al., 2015; Zhao et al., 2016). Fifteen HDACs were characterized in maize (*Zea mays*), including 10 members of the RPD3/HDA1 family, one member of the SIR2 family, and four members of HD2 family (Hu et al., 2011). It was reported that ZmHDACs might participate in cold stress responses by selectively regulate the transcription of cold-responsive genes (Hu et al., 2011). Twenty-eight HDACs were identified in Soybean (*Glycine max*), including six members in the HD2 family, four members in the SIR2 family, and 14 members in the RPD3/HDA1 family. Genome-wide RNA-seq analysis indicated that GmHDACs might be involved in the gene regulation during flower initiation (Liew et al., 2013). The grape (*Vitis vinifera*) genome contains 13 *HDAC* genes (Aquea et al., 2010). Eleven HDACs were characterized in litchi (*Litchi chinensis* Sonn. cv. Feizixiao), which might play important roles in fruit abscission (Peng et al., 2017). In addition, 15 HDACs were identified in tomato (*Solanum lycopersicum*), which might be involved in gene regulation during reproductive development (Zhao L. M. et al., 2015).

Histone deacetylases can contribute to the establishment of epigenetic states and mediate the crosstalk of histone acetylation with other histone modifications. Our previous studies revealed that HDA6 interacts with the DNA methyltransfrase MET1 to regulate DNA methylation and histone deacetylation in *Arabidopsis* (Liu et al., 2012). In addition, HDA5 and HDA6 form a protein complex with the histone demethylase FLD, suggesting that regulatory crosstalk between histone demethylation and deacetylation through the direct interaction between HDA5/HDA6 and FLD (Yu et al., 2011; Luo et al., 2015). Furthermore, HDA6 also interacts with histone methyltransferases SUVH4/5/6 and they function collaboratively in transposon silencing by removing the acetyl group from histone H3 and adding the methyl group to histone H3K9 (Yu et al., 2017). Furthermore, a specific acetylation site, H3K14, which is associated with transcription activation, is identified to be propionylated and butyrylated *in vivo*, suggesting histone acetylation and other modifications may acts in combination to modulate chromatin condensation and transcription outputs (Kebede et al., 2017).

## Functions of HDACs in Salt and Drought Stress Responses in *Arabidopsis*

**FIGURE 1:**
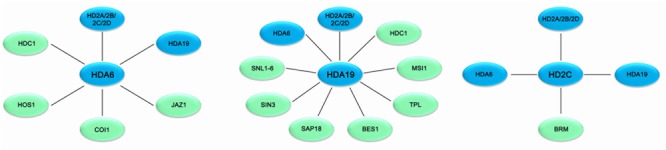
Association networks of HDACs and interacting proteins in plant stress response in *Arabidopsis*. HDACs are associated with various protein involved in stress response in *Arabidopsis*. Solid bars indicate physical interaction between HDACs (blue) and other protein (green).

Recent studies indicated that HDACs play important roles in plant abiotic stress responses (**Table 1**). In *Arabidopsis*, members of the RPD3/HDA1 family were found to interact with various proteins involved in plant stress responses and regulate gene expression through histone deacetylation (**Figure 1**). HDA6 is involved in drought stress tolerance by regulating gene expression in the acetate biosynthesis pathway (Kim et al., 2017). HDA6 also regulates the jasmonate (JA) associated stress response by interacting with COI1 and JAZ1, the key regulators of JA signaling (Devoto et al., 2002; Zhu et al., 2011). *hda9* mutant plants showed an enhanced tolerance to salt and drought stress. A large number of stress response genes, especially water deprivation stress-related genes, were up-regulated and hyper-deacetylated in *hda9* mutants, indicating that HDA9 may act as a negative regulator in modulating stress responsive gene expression through histone deacetylation (Chen et al., 2016; Kim et al., 2016; Zheng et al., 2016).

**Table 1:** Plant HDACs in abiotic stress responses.

Species	HDACs	Target genes	Histone substrates	Abiotic stress	Reference
*Arabidopsis*	HDA6	*ABI1, ABI2*	H3K9K14ac	ABA and salt	Chen and Wu, 2010; Chen et al., 2010; Luo et al., 2012b,c
		*ABA1, PYL4, DR4, FLC, EIN3/EIL1*	H3K9K14ac	ABA, salt, cold, and drought	Devoto et al., 2002; Zhu et al., 2011; Jung et al., 2013; Perrella et al., 2013; Kim et al., 2017
	HDA9	*AtLIP3, AtPAD3, AtLTP6*	H3K9ac	ABA and salt	Zheng et al., 2016
	HDA19	*AtERF7*	–	ABA	Song et al., 2005
		*ERF3, ERF4*	–	Salt	Song and Galbraith, 2006
		–	–	–	Luo et al., 2012b,c
		*ABA1, PYL4, DR4*	H3K9K14ac	ABA, salt, and drought	Perrella et al., 2013
		*CYP707A1, CYP707A2*	H3K9K18ac	ABA	Wang et al., 2013
		*ABI3*	H3K9K14ac	ABA	Ryu et al., 2014
		*PYL4, PYL5, PYL*	H3K9ac	ABA	Mehdi et al., 2016
	HD2A	–	–	ABA and salt	Luo et al., 2012b,c
	HD2B	–	–	ABA and salt	Luo et al., 2012b,c
	HD2C	*ABI1, ABI2*	H3K9K14ac	ABA, salt, and heat	Luo et al., 2012b,c;Buszewicz et al., 2016
	HD2D	–	–	ABA and salt	Luo et al., 2012b,c
		–	–	Drought, salt, and cold	Han et al., 2016
Rice	HDA701,	–	–	Salt	Hu et al., 2009
	HDA702,				
	HDA704,				
	HDA705,				
	HDA706,				
	HDA712,				
	HDA714,				
	HDA716,				
	HDT701,				
	HDT702,				
	HDA709,				
	SRT702				
	HDT701, HDT702	–	H4ac	Salt	Zhao J. H. et al., 2015
	HDA705	–	–	ABA and salt	Zhao et al., 2016
	SRT701,	–	H3K9ac	–	Zhong et al., 2013
	SRT702				
Tomato	SlHDA1-SlHDA9	–	–	High/low temperature, salt, and dehydration	Guo et al., 2016
Maize	ZmHDAC1,	*ZmDREB1*	H3K9ac,	Cold	Hu et al., 2011
	ZmHDAC2,		H4K5ac, and H4ac		
	ZmHDAC3,				
	ZmHDAC6,				
	ZmHDAC8,				
	ZmHDAC110				
Common bean	PvHDA6	–	–	Cold	Hayford et al., 2017
Barley	HvHDAC2-1, HvHDAC2-2	–	–	ABA, JA, and SA	Demetriou et al., 2009

HDA19 is recruited by the AP2/EREBP transcription factor AtERF7 and forms a repressor complex with AtSin3 to repress transcription of stress response genes in ABA and drought stress response (Song et al., 2005). Furthermore, HDA19 interacts with SNL1 and forms a repressor complex with SNL2 to regulate ABA synthesis through histone deacetylation of the specific target genes (Wang et al., 2013). In addition, HDA19 is associated with BES1 and TPL and the BES1-TPL-HDA19 repressor complex modulates *ABI3* expression through histone deacetylation (Ryu et al., 2014). HDA19 was also found to act in a protein complex with the WD40-repeat protein MSI1 and the SIN3-like proteins to fine-tune ABA signaling in *Arabidopsis* (Mehdi et al., 2016). Both MSI1 antisense and HDA19 RNAi plants showed enhanced tolerance to salt stress treatment. The ABA sensitivity of gene expression is modulated through the MSI1-HDA19-SIN3 complex via modulating the transcription of ABA receptor genes (Mehdi et al., 2016). In addition, both HDA6 and HDA19 form a protein complex with HDC1 required for deacetylation of H3K9K14 in the plant abiotic stress responses (Perrella et al., 2013).

HD2-type proteins are plant specific HDACs and have been characterized in *Arabidopsis*, rice, soybean, barley tomato and maize (Demetriou et al., 2009; Luo et al., 2012b; Kim et al., 2015). The expression of *Arabidopsis HD2A, HD2B, HD2C*, and *HD2D* is repressed by ABA and high salt treatment (Luo et al., 2012b). Overexpressing *HD2D* in transgenic *Arabidopsis* resulted in increased tolerance to drought and salt stresses, suggesting that HD2D is involved in environmental stress responses (Han et al., 2016). Furthermore, overexpression of *HD2C* enhances salt and drought tolerance by modulating ABA-responsive genes (Sridha and Wu, 2006). HD2C functionally associates with HDA6 and regulates ABA-responsive gene expression through histone deacetylation (Luo et al., 2012b).

## Functions of HDACs in Temperature Stress Responses in *Arabidopsis*

HDA6 plays a critical role in cold tolerance by regulating the expression of cold stress responsive genes (To et al., 2011; Kim et al., 2012; Jung et al., 2013). Moreover, the cold signaling attenuator, HOS1, interacts with HDA6 and inhibits the binding of HDA6 to *FLC* chromatin, resulting in a delayed flowering under short-term cold stress (Jung et al., 2013).

Overexpressing *HD2D* in transgenic *Arabidopsis* resulted in increased tolerance to cold stresses (Han et al., 2016). HD2C acts as a negative regulator of heat-activated genes in plants exposed to heat treatment through interacting with the chromatin remodeling factor BRAHMA (BRM) (Buszewicz et al., 2016). HD2A, HD2C, and HD2D can interact with both HDA6 and HDA19 (Luo et al., 2012c), suggesting that HD2-type HDACs functionally associate with RPD3-type HDACs in the multiprotein complex to regulate stress response genes in plants.

Additionally, the HDAC inhibitor trichostatin A (TSA) treatment can lead to an increased ROS level in animal cells (Sun et al., 2014). Similarly, an increased ROS level and enhanced antioxidant activity were detected by TSA treatment in *Arabidopsi*s, suggesting that HDACs may be involved in the regulation of the ROS content under stress conditions in *Arabidopsi*s (Jadko, 2015). Moreover, total HDAC activity was reduced by GSNO and *S*-nitroso-*N*-acetyl-DL-penicillamine treatment in plants (Mengel et al., 2017). ChIP-seq analysis revealed that the global H3K9/14ac was affected by NO treatment, resulting in the hyperacetylation of stress-related genes (Mengel et al., 2017).

## Functions of HDACs in Abiotic Stress Responses in rice and Other Plant Species

In rice, microarray analysis revealed that the expression of *OsHDA703* and *OsHDA710* was induced, whereas the expression of several other *HDAC* genes was repressed under high salt and drought treatment (Hu et al., 2009). In addition, the expression of *OsHDT701* and *OsHDT702* was affected under ABA, salt, and PEG stresses (Zhao J. H. et al., 2015). Interestingly, *OsHDA709* and *OsSRT702* were induced by drought treatment, but repressed by high salt. Genome-wide acetylation and binding analysis indicated that *OsSRT701* may directly regulate the expression of stress-related genes by H3K9 deacetylation (Zhong et al., 2013). Overexpressing of *OsHDT701* in transgenic rice resulted in increasing tolerance to salt and drought, and enhanced resistance to both *Magnaporthe oryzae* and *Xanthomonas oryzae* pv. *oryzae (Xoo)* (Ding et al., 2012; Zhao J. H. et al., 2015). In contrast, overexpression of *OsHDA705* in transgenic rice resulted in a decreased salt and ABA stress resistance during seed germination (Zhao et al., 2016). In addition, transcription of ABA biosynthetic genes is increased in the *HDA705* overexpressing transgenic plants, suggesting that HDA705 may play vital roles in response to abiotic stresses in rice (Zhao et al., 2016).

In common bean (*Phaseolus vulgaris* Linn.), the expression of *PvHDA6* was increased during cold treatment, indicating *PvHDA6* is a cold response gene involved in regulation of plant abiotic stress tolerance (Hayford et al., 2017). In tomato (*Solanum lycopersicum*), *SlHDACs* were induced in various degrees under high salinity, dehydration, and different high/low temperature treatments, suggesting that *SlHDACs* might function in different stress responses (Guo et al., 2016). Treatment with the HDAC inhibitor, suberoylanilide hydroxamic acid (SAHA), enhanced plant salinity stress tolerance, indicating that HDACs might function in salt stress tolerance in *cassava* (Patanun et al., 2017). In barley (*Hordeum vulgare*), the transcription of *HvHD2* genes were affected by multiple plant hormones, such as ABA, salicylic acid, and JA (Demetriou et al., 2009). In maize (*Zea mays*), the transcriptional patterns of *ZmHDACs*, including *ZmHDAC1, ZmHDAC2, ZmHDAC3, ZmHDAC6, ZmHDAC8*, and *ZmHDAC110* was altered in response to low temperature. Levels of histone H3K9ac, H4K5ac, and H4ac were decreased after cold treatment. In addition, the expression of *ZmDREB1* and *ZmCOR413* was repressed by trichostatin A (TSA) treatment under cold stress conditions. Chromatin immunoprecipitation assays suggested that ZmHDACs may directly activate the expression of *ZmDREB1* through histone deacetylation (Hu et al., 2011).

## HDACs and Autophagy

Autophagy is a tightly regulated pathway involving the lysosomal degradation of cytoplasmic organelles or cytosolic components. This pathway can be stimulated by multiple forms of cellular stress. Recent studies indicated that HDA9 may repress the expression of a number of autophagy related genes including *APG9, ATG2, ATG13*, and *ATG8e* in *Arabidopsis* (Chen et al., 2016). However, the molecular mechanism how HDACs participate in autophagy remains largely unknown in plants.

Involvement of protein acetylation in autophagy was widely studied in mammalian and yeast cells (Yi et al., 2012; Fullgrabe et al., 2013; Huang et al., 2015; Su et al., 2017). In *Saccharomyces cerevisiae*, Rpd3 is required for K9 and K18 acetylation/deacetylation of Autophagy-related 3 (Atg3) during autophagy (Yi and Yu, 2012; Yi et al., 2012). In mammals, Atg8 is deacetylated by Sirt1 in response to starvation, and overexpression of Sirt1 can stimulate autophagosome formation (Lee et al., 2008). Deacetylation of LC3, a key initiator of autophagy, at K49, K51 by Sirt1 is essential for starvation-induced autophagosome formation in human cells (Huang et al., 2015). The acetylation or deacetylation of LC3 mediated by Sirt1 is linked to its nucleocytoplasmic transport. Under normal conditions, both nuclear and cytoplasmic forms of LC3 are acetylated (Huang and Liu, 2015). However, in response to starvation, LC3 is redistributed from the nucleus to the cytoplasm. In addition, deacetylation levels of LC3 is required for the interaction between LC3 and Atg7 (Huang and Liu, 2015; Huang et al., 2015). Taken together, these data suggested that HDACs act as regulators of autophagy in mammalian and yeast cells. Further research is required to determine how plant HDAC functions in this process.

## Conclusion and Future Prospects

Histone deacetylases are recruited by diverse DNA-binding transcriptional factors forming multiple protein complexes to fine-tune the chromatin structure and modulate the gene expression in plant responses to environment stresses. Identifying the key components of HDAC complexes though yeast two-hybrid screening and *in vivo* immunoprecipitation in combination with mass spectrometry (IP-MS) is indispensable for understanding the functional organization of protein-protein interaction networks in the regulation of abiotic stress responses. To further understand how HDACs are involved in plant responses to abiotic stress, it is also important to identify the transcriptional regulatory network and the genome-wide binding site of HDACs by using RNA-seq and ChIP-seq approaches.

Recent studies indicated that non-histone proteins can also be acetylated or deacetylated by HATs or HDACs (Kovacs et al., 2005; Tran et al., 2012; Hao et al., 2016). For instance, *Arabidopsis* HDA6 can enhance brassinosteroid (BR) signaling by directly deacetylating the GSK3-like kinase BR-INSENSITIVE 2 (BIN2) and inhibits its kinase activity in the BR signaling pathway (Hao et al., 2016). In addition, the NAD+-dependent HDAC SRT1 could also remove the lysine acetylation from *Arabidopsis* c-Myc-Binding Protein-1 (AtMBP-1) and significantly enhance its stability in regulating primary metabolism and stress response (Liu et al., 2017). Further research is required to investigate acetylation and deacetylation of non-histone proteins in plant abiotic stress response. Environmental stresses cause significant crop losses on an annual basis. Uncovering the function of HDACs in plant responses to abiotic stress will contribute to our understanding of how plants adapt to environmental changes, which will be applicable in improvement of agricultural productivity.

## Author Contributions

ML and KW conceived the idea. ML and KC wrote the manuscript. KW revised the manuscript. ML, KC, YX, SY, and KW critically evaluated the manuscript. All authors read and approved the manuscript.

## Conflict of Interest Statement

The authors declare that the research was conducted in the absence of any commercial or financial relationships that could be construed as a potential conflict of interest.
